# Patients’ satisfaction with dental care: a qualitative study to develop a satisfaction instrument

**DOI:** 10.1186/s12903-018-0477-7

**Published:** 2018-01-30

**Authors:** Jennifer Yu Ning Luo, Pearl Pei Liu, May Chun Mei Wong

**Affiliations:** 1 Dental Public Health, Faculty of Dentistry, The University of Hong Kong, 34 Hospital Road, Sai Ying Pun, Hong Kong; 2 Department of Stomatology, The Yiwu Hospital Affiliated to Wenzhou Medical College, 699 Jiangdong Road, Yiwu, Zhejiang Province China

**Keywords:** Dental care, Satisfaction, Outcome assessment, Qualitative study

## Abstract

**Background:**

To explore and better understand how patients evaluate satisfaction in dental care and elicit information from them to develop a dental satisfaction instrument.

**Methods:**

Patients currently receiving dental treatment in a teaching hospital were invited to be part of a qualitative research project which involved focus group discussion. Focus groups were conducted in Cantonese and discussions were recorded (audio and video) and later transcribed.

**Results:**

Thirty patients participated and a thematic analysis of data from four focus groups helped generate a questionnaire on dental satisfaction. Six themes were extracted from the contents of the focus group: (i) attitude, (ii) cost, (iii) convenience, (iv) pain management, (v) quality, and (vi) patients’ perceived need for prevention of oral disease. Compared to the existing Dental Satisfaction Questionnaire (DSQ), majority of the dental satisfaction aspects mentioned in focus group discussions were similar to items in DSQ supporting its content validity. Focus groups covered more aspects including attitude of dental supporting staff, convenience of emergency services, admission of patients and treatment duration. Consideration of the clinical skills of the operator, hospital infection control, and knowledge on prevention of oral disease were also expressed.

**Conclusions:**

The focus group discussions elicited the views of patients not covered by DSQ items thereby suggesting areas for development of a new satisfaction questionnaire.

## Background

Patient satisfaction is crucial in the evaluation of the overall quality of care, and thus the improvement of care services as it may be considered as an outcome of dental care in addition to clinical outcomes [1].

As a patient-reported-outcome, data on satisfaction can be collected using a quantitative or qualitative approach. In the dental field, various quantitative questionnaires, such as the 19-item Dental Satisfaction Questionnaire (DSQ) [2], the 10-item Dental Visit Satisfaction Scale (DVSS) [3], the 22-item Scale for Measuring Consumer Perception of Service Quality (SERVQUAL) [4], and the 31-item Australian Dental Satisfaction Scale (DSS) [5], have been developed to assess patients’ satisfaction level. DVSS was developed from a medical interview satisfaction scale with the items transferred to dentistry. The predecessor of SERVQUAL was a commercial questionnaire to evaluate customer satisfaction with the service. The statements used in Australian DSS were also based on the content of existing satisfaction scales on medical care. Even though the procedure for constructing these questionnaires include illuminating the dimensions along with interviewees’ (e.g. consumers and patients) perception by qualitative research at the initial stage and have also been tested for reliability and validity in populations with dental problems [6–8]. However, none of the questionnaires were developed from the outset by qualitative research in a dental population. There is a need to conduct qualitative research among the dental population to explore the items that represent satisfaction in the specific field of dental services.

DSQ is a well-developed 19-item questionnaire recognized for covering multi-dimensional constructs of satisfaction classified into 6 scales (Access, Availability/Convenience, Cost, Pain, Quality and Continuity) and a global access scale (General satisfaction). DSQ was derived using the data from the National Health Insurance Study in United States. This was conducted amongst adults who enrolled in an insurance plan covering most dental services except orthodontics. The final scale was constructed using factor analysis and tested for reliability and validity in different populations [9, 10]. Similar to the questionnaire development process mentioned above, this scale lacks qualitative input at the stage of questionnaire construction.

The Chinese version of the 19-item DSQ has been used in Hong Kong in a telephone survey on the general public’s satisfaction towards dental care. However, not all scales reported good internal consistency (Cronbach’s alpha: 0.39–0.84) and test-retest reliability (correlation coefficients: 0.46–0.85) [11]. In another study, DSQ was modified and was used in a survey assessing patients’ satisfaction towards dental services provided by a university dental clinic [12]. The modified version had additional 4 items to the traditional DSQ so as to cover the influence of dental team members on patients’ satisfaction rather than dentist performance alone. No reliability nor validity has been reported for this modified DSQ. In 2010 and 2012, two surveys were conducted to assess the satisfaction of patients receiving care in a teaching dental hospital in Hong Kong using the modified 23-item DSQ. These surveys reported that the internal consistency of DSQ was low (Cronbach’s alpha < 0.50), except for the domain “Quality” (Cronbach’s alpha > 0.70). The domains with the lowest internal consistency were “Access” and “Availability” (Cronbach’s alpha ≤0.30) [unpublished data]. The low internal consistency may be contributed by the cultural differences in evaluating dental satisfaction, which may also affect the validity of the instrument to be used locally. Thus it is important to evaluate the content validity of DSQ.

Content validity means the extent to which an instrument measures the concept it is intended to measure, i.e.*,* for an instrument that measures patient satisfaction, does this instrument cover all aspects related to patient satisfaction comprehensively and if the instrument can be fully understood and accepted by respondents [13]. The most appropriate way to collect data to support content validity is by conducting qualitative research. This helps to obtain reports on the status of a patient’s health condition, perceptions and experiences directly from the patient, without the interpretation of the patient’s response by a clinician or anyone else [14]. This approach has not been used when DSQ was developed. There is a need to construct the items which reflect the content validity by qualitative study.

The aim of this study was to elicit information from qualitative interviews with patients to improve the psychometric properties of a dental satisfaction instrument.

## Methods

By using the qualitative approach, the patient experience and satisfaction level towards dental care were collected across four focus groups in Prince Philip Dental Hospital (PPDH), the teaching dental hospital of The University of Hong Kong. The research question was “What were the aspects that patients usually considered when evaluating the satisfaction of dental care?” Ethical approval was obtained from the Institutional Review Board of The University of Hong Kong/Hospital Authority Hong Kong West Cluster (HKU/HA HKW IRB, Reference number: UW 14–200). The study was conducted in full accordance with the World Medical Association Declaration of Helsinki.

### Subjects

The participants were patients who had received or are receiving dental treatment at PPDH. The inclusion criteria were Cantonese speaking patients aged 18 years and above. The exclusion criteria were those with communication difficulties. In order to acquire a sample with different categories of dental problems and treatments, we formed focus groups based on a list of patients with dental appointments scheduled from different clinical disciplines. The eligible patients were contacted and invited to participate in the focus group discussion by a research assistant using the telephone numbers obtained from the patients’ folders. Detailed information including the purpose, procedure, and significance of conducting this study were explained to the patients during the phone calls. Written consent was obtained when they attended the focus group discussion.

### Data collection

A topic guide for the focus group discussion was developed around the research question. It provided a framework for structured discussions and stimulation of the interaction between the researcher and the participants, as well as among the participants. However, participants could also raise issues outside the framework that they considered to be important. The topic guide was developed from the Chinese version of modified DSQ used in the patient satisfaction survey in PPDH in 2012.

The main topics were the experience of receiving dental care in PPDH, patients’ most favorable or unfavorable aspects of the dental services, the aspects they usually considered when asked on satisfaction level, e.g. what aspects they thought “Access” and “Availability” should cover, why they were or were not satisfied. The participants were also asked to provide some suggestions to improve the dental services.

The focus group discussions were scheduled on the same days of the participants’ dental clinic appointments. The discussion were conducted in Cantonese and each group discussion lasted about one hour. One facilitator (CM Wong, one of the authors) facilitated the discussion and encouraged the participants to express their opinions and views freely. The discussions were video and audio taped and later transcribed for analysis.

### Data analysis

The audio and video recording from the focus group discussions were transcribed *verbatim* in Chinese, together with non-verbal communicative behaviors between participants added in the transcripts. Thematic content analysis was performed [15]. The main themes and sub-themes emerged from the data. The transcript were reviewed by two analysts (YN Luo & P Liu, authors) and meaningful “text units” were extracted manually through line-by-line coding. A unified “pre-set” coding framework was developed when coding on the transcription of the first focus group and the thematic interrelation was discussed to attain agreement. To ensure the inter-coder agreement, the analysts continued to code the other three transcriptions using this framework and the “emergent” codes were discussed, compared and harmonized afterwards. Ultimately, the refined final version of the codes were applied to the total transcriptions independently one more time and agreement was attained between two coders. Quotations were selected to illustrate the observed themes and sub-themes. The items/statements were then derived by the authors generalized from the selected quotations.

## Results

### Sample characteristics

A total of 65 patients were contacted by telephone and invited to participate and 30 patients were recruited successfully. Four focus groups with two consisting of 6 subjects and another two consisting of 9 subjects were conducted between April and May 2014. There were 15 females and 15 males and the range of their ages was 18 to 86 years old (mean 50.1, SD 18.0). Eighteen patients had been receiving multiple treatments in PPDH and twelve of them only received a single treatment. The treatments received by the participants included endodontic treatment, periodontal treatment, orthodontic treatment, dental implant, prosthesis, tooth extraction and oral maxillofacial surgery (Table 1).

**Table 1:** Characteristics of the participants

		Number
Gender		
Male		15
Female		15
Age (in years)		
18–30		6
31–50		8
51–60		6
61–70		7
> 70		3
Treatment		
Single treatment	Endodontic treatment	1
	Periodontal treatment	2
	Orthodontic treatment	3
	Dental implant	2
	Tooth extraction	1
	Oral maxillofacial surgery	3
Multiple treatment		18

### Themes and subthemes on dental satisfaction

Various opinions on dental satisfaction arose from the group discussion. They were organized by key emergent themes on dental satisfaction derived from patients’ consideration when they assess dental services. Data saturation was achieved when no more new aspects emerged when conducting the fourth (and last) focus group discussions.

Six themes were extracted from the content of the focus group discussions on dental satisfaction. According to the order from the conversation, they included (i) attitude, (ii) cost, (iii) convenience, (iv) pain management, (v) quality, and (vi) patients’ perceived need for prevention of oral disease.

### Attitude

“Attitude” was always the first aspect mentioned by the participants when asked about their satisfaction level towards the dental services. This included attitude of the dentists (operators) as well as other dental support staff, such as dental surgery assistants (DSA). The key considerations of good dental services were the level of details of the interpretation to the patients on the disease itself, the treatment process and complications; the attention and concern given to the patients throughout the course of treatment; the kind words of comfort and encouragement given to the patients in the process of treatment.

*“I think the dentists or students are careful. They explained clearly to me about the treatment purpose and procedure when I asked for more information. They told me what they were doing, what they were going to do, when to do certain treatment, all was very clear. And before I received dental implant treatment, they had solved all the other problems in my oral cavity and had ensured that no more problems would affect the dental implant.”*

*(Participant 25, Group 4, female, multiple treatments)*

*“When doing the scaling, the dentists in PPDH gave me instructions of how to take care my own teeth while those in private clinic did not. They only provided scaling and actually I did not know what they did for me. In PPDH, they showed me what my oral problem was with a mirror and explained to me.”*

*(Participant 19, Group 3, male, multiple treatments)*

*“The dentist who extracted my teeth and his DSA were so kind that they kept following up my situation as I suffered pain and my cheek was badly swollen after extraction. They always comforted me and explained clearly to me what I could do and I thanked them for their cares. ”*

*(Participant 1, Group 1, female, multiple treatments)*

When detailed explanation was considered as an important aspect of “attitude”, some participants believed that the dentists should give more information about the treatment procedure.

*“Sometimes the dentists’ explanation about the treatment was not detailed enough, because I was going to receive maxillofacial surgery, I expected the dentist to explain more in detail. I wanted to know about the length of the surgery, the possible complications and risks.”*

*(Participant 3, Group 1, female, single treatment)*

The attitude of DSA when cooperating with the dentist also affected the satisfaction of the participants.

*“Sometimes, during the treatment, the DSA spoke too much and kept chatting with the dentist, which distracted the dentist’s attention and made him not focused enough.”*

*(Participant 12, Group 2, male, multiple treatments)*

### Cost

Cost was mentioned by almost all participants in the consideration of the satisfaction level towards the dental care provided by this hospital. They agreed that the price of the dental care services provided by this hospital was much lower compared with private clinics.

*“I feel quite worthwhile to enjoy the dental care service at such a low price. I believe if I visited a dentist in private setting, I can’t afford the same dental care service. I used to receive an endodontic treatment in private clinic that cost me 8000-9000 Hong Kong dollars while it only cost me dozens of dollars for each visit here in PPDH. I think it is quite worthwhile.”*

*(Participant 11, Group 2, male, multiple treatments)*

*“The price is much cheaper in PPDH, for example, the price of dental implant in private clinic is three times as high compared with the price in PPDH.”*

*(Participant 16, Group 3, male, multiple treatments)*

### Convenience

When the participants were asked on the unfavorable aspects of the hospital service, the theme “convenience” emerged, which included accessibility (e.g., opening hours, location of the hospital, appointment booking, admission of patients, emergency service) and the time spent in solving the teeth problems in the hospital. Some of the segments on the “accessibility” are as follows:

*“The hospital is closed on Saturday and Sunday, which makes a lot of patients have to ask for leave from work in order to visit the dentist.”*

*(Participant3, Group1, female, single treatment)*

*“It is more difficult to book an appointment in PPDH compared with private clinic. Not every phone call would be answered, and sometimes you need to leave a message and wait for response.”*

*(Participant 5, Group 1, male, single treatment)*

A big concern related to convenience was the difficulty of getting admitted as a patient in this teaching hospital. Not all patients would be admitted if they did not meet the requirements of teaching cases. After screening, the waiting time for the appointment to be attended by a dentist could be long. Furthermore, this dental hospital did not provide emergency services.

*“I am not the only one who tried to get admitted to the hospital for receiving the dental care and I waited almost one year for my first appointment. Some of my friends also wanted to receive dental care in PPDH as they needed some dental care, however, they failed to get admitted. It would be better if more people could get admitted.”*

*(Participant 20, Group 3, female, multiple treatments)*

*“When something emergency happened, they told me to visit the dentists in private clinics, which made me feel no sense of belonging.”*

*(Participant 4, Group 1, male, multiple treatments)*

The time spent on solving the dental problems in the hospital had also become one of the reasons affecting patients’ satisfaction level, which included waiting time at the clinic, duration of the entire treatment process (treatment duration) and the duration of travel for each dental visit.

*“I don’t like the treatment schedule, it was too long between each visit, sometimes I had to wait for one month and sometimes I waited for 2 to 3 months (before I can see the dentist again).”*

*(Participant 8, Group 2, male, single treatment)*

*“I think the treatment duration should be shortened so that I do not need to visit the dentist for so many times.”*

*(Participant 6, Group 1, female, multiple treatments)*

*“During the treatment, sometimes the students needed the dentist (clinical teacher) to come and check before they can take the next step, which kept me waiting for a long time, especially when the dentist was teaching other students, I had to keep my mouth wide open and wait.”*

*(Participant 15, Group 2, female, single treatment)*

### Pain management

Pain management was another aspect mentioned by the participants when they talked about the satisfaction. The strategy of pain management in the process of treatment (e.g., intra-operative anesthesia) and the use of post-operative analgesics affected their satisfaction with the dentist.

*“I think they did well in pain management and they never let me feel very painful. I could raise my hand and he would inject more anesthetic before carrying on, thus I felt no pain.”*

*(Participant 2, Group 1, male, single treatment)*

*“During the treatment such as deep scaling (subgingival scaling), even though six shots of local anesthetics were injected, I still felt painful… My teeth was hypersensitive, the cold water irritated my teeth and made me feel really painful.”*

*(Participant 17, Group 3, male, single treatment)*

*“Because of the inflammation, … the dentist prescribed me a stronger pain killer, but not everyone could take that pain killer, I got stomachache after taking it for two times.”*

*(Participant 1, Group 1, female, multiple treatment)*

### Quality

When the participants were probed on the definition of the “quality” of dental services, the participants mentioned technology, equipment and facilities, perceived skill of the dentists, disinfection, manpower and improvement of oral health condition after treatment.

*“I considered the technology used in treatment when evaluating the quality of dental care. I don’t think the dentists in private clinic use the same technology as in this hospital.”*

*(Participant 5, Group 1, male, single treatment)*

*“I think the equipment is good enough in PPDH, the X-ray can take picture in 360 degrees (oral panoramic radiography) and I do not need to move at all. I think the equipment here is much better than that in private clinics.”*

*(Participant 16, Group 3, male, multiple treatments)*

Some participants expressed concern towards the skill of students whereas some participants expressed more confidence.

*“When that student examined my teeth, … I felt that his skill was not so good, he made me feel painful.”*

*(Participant 29, Group 4, male, single treatment)*

*“Even though the master student had to have a professor to supervise and instruct him throughout the treatment procedure. I still felt confident about his skills. ”*

*(Participant 27, Group 4, female, multiple treatments)*

The strict disinfection was also a consideration for patients to assess the quality.

*“What I appreciate the most is the disinfection in PPDH. Used stuff was put into plastic bags. And the DSA in charge of disinfection recycled all the used stuff for disinfection and sterilized stuff was prepared for the next patient.”*

*(Participant 21, Group 3, female, single treatment)*

However, insufficient manpower and not receiving follow-up from the same dentist were aspects affecting the quality of dental care as some participants mentioned:

*“I saw the DSA was helping a student for a while then she said that she needed to help another student and left, thus the student had to wait for her to come back and continue the help… I saw that there was a shortage of DSA…”*

*(Participant 15, Group 2, female, single treatment)*

*“I saw the student had no DSA to help him, and he was busy doing all the things by himself. I think maybe sometimes he forgot to change the gloves because he was too busy.”*

*(Participant 7, Group 2, female, single treatment)*

*“My orthodontic treatment required a long time, at the beginning I was under the care of one dentist and then changed to another later on.The communication between dentists was not good enough, the second dentist did not know my case very well.”*

*(Participant 5, Group 1, male, single treatment)*

The improvement of post-treatment oral health was also a consideration for patients assessing the “quality”:

*“The treatment outcome was good in this hospital. I received the treatment ten years ago, and I have been free from dental problem for a long time since then… Thus I believe the quality of the dental care in PPDH is quite good.”*

*(Participant 30, Group 4, female, multiple treatments)*

### Patients’ perceived need for oral disease prevention

A considerable number of patients expressed their appreciation of receiving knowledge on prevention of oral disease when visiting a dentist or dental student in PPDH.

*“(One aspect I am happy with is) the oral health education given here.”*

*(Participant 20, Group3, female, multiple treatments)*

In addition, they hoped to have a standard regular follow-up and referral system, thus they could receive some regular checkups for early prevention or detection of dental problems.

*“I am wondering whether more effort can be put in oral health education… because I know little knowledge of taking care of my teeth… I think promotion via TV can be used to educate people regarding the importance of oral health care.”*

*(Participant 10, Group 2, female, single treatment)*

*“I hope I can book appointment for regular checkup in PPDH after finishing the treatment.”*

*(Participant 13, Group 2, male, multiple treatments)*

Based on the above findings, the total 9 new items not covered by the DSQ-19 were emerged in the focus group discussions. New items derived from the focus group discussions according to the themes and sub-themes are summarized in Table 2.

**Table 2:** Themes, subthemes and new items derived from focus groups

Themes	Sub-themes	New Items (Statement)	DSQ Items
Attitude	Attitude of the dentists/students		Item 2. Dentists are very careful to check everything when examining their patients (Quality) Item 11. Dentists aren’t as thorough as they should be (Quality) Item 16. Dentists usually explain what they are going to do and how much it will cost before they begin treatment (Quality) Item 6. Dentists always treat their patients with respect (Quality)
	Attitude of the dental supporting staff	1. The working attitude of the nurse made me feel uncomfortable	
Cost			Item 3. The fees dentists charge are too high (Cost)
Convenience	Access	2. The dental service is accessible when I need emergency dental care	Item 15. Office hours when you can get dental care are good for most people (Access) Item 9. Places where you can get dental care are very conveniently located (Availability/Convenience) Item 13. It’s hard to get an appointment for dental care right away (Access) Item 5. Peoples are usually kept waiting for a long time when they are at the dentist’s office (Access)
		3. The admission procedure of the Hospital is convenient and fast	
	Treatment procedure	4. There are too many visits throughout the whole treatment plan	
Pain management			Item 8. Dentists should do more to reduce your pain (Pain management)
Quality	Operator(s)	5. I am confident about the clinical skills of the dentists/students	Item 14. Dentists are able to relieve or cure most dental problems that people have (Quality) Item 7. There are enough dentists around here (Availability/Convenience) Item 12. I see the same dentists just about every time I go for dental care (Continuity) Item 18. Dentist’s offices are very modern and up to date (Environment)
	Hospital	6. The infection control procedures are satisfactory	
Patients’ perceived needs for oral disease prevention	Oral health education	7. The dentists/students have delivered me instructions of how to take care my own teeth. (appropriate oral health education)	
		8. I can easily obtain oral health information from the hospital	
	Regular follow-up	9. Adequate post-treatment follow-up is provided	

Five items in DSQ-19 that were not emerged in the focus group discussions:

Item 10. Dentists always help you to avoid unnecessary expenses (Cost)

Item 4. Sometimes you avoid going to the dentist because it is so painful (Pain management)

Item 19. You are not concerned about feeling pain when you go for dental care (Pain management)

Item 17. Dentists should do more to keep people from having problems with their teeth (Quality)

Item 1. There are things about the dental care I receive that could be better (General satisfaction)

## Discussion

### Summary of findings

Patient satisfaction is crucial in the evaluation of the overall quality of care, and thus the improvement of care services. This study is a first step to build up a satisfaction assessment tool and the items from this study would promote the reliability and validity of existing questionnaires which will assist the dental care system in providing optimal and relevant care to patients. The qualitative study identified several previous themes in validated DSQ questionnaire, as well as concepts covered under different themes. Five similar themes in the DSQ: (i) attitude, (ii) cost, (iii) convenience, (iv) pain management, (v) quality, and one theme not mentioned in the DSQ i.e., (vi) patients’ perceived needs for oral disease prevention. There are a total of 9 new items derived by focus group not covered in DSQ which involved in four aspects (subthemes), namely the attitude of the dental supporting staff; convenience of the dental service which includes accessibility of emergency services and the hospital admission procedure, plan of the whole treatment procedure; quality including the clinical skills of the operator and hospital infection control; and patients’ perceived needs for oral disease prevention.

### Significance of this study

This qualitative study can be used to understand a patient-reported outcome (PRO) concept, inform decision making related to instrument design and demonstrate the content validity of a measure, thus benefitting instrument design and selection [16]. Focus groups can help better understand how subjects express their feelings and what the perceptions and opinions of the subjects are. This happens via creating a more natural environment and stimulating interaction between subjects [17]. The total 9 questions formulated through this study will be used to generate a modified questionnaire to be used in a satisfaction survey later (Table 2, items with asterisk mark). The survey (quantitative study) will test the reliability and validity of DSQ in assessing patient satisfaction with dental care based on the new version, ascertain how well they are currently performing on those items important to patients. Secondly, it is helpful for a hospital to target service improvements in those aspects of care that were important to patients.

### Strengths and limitations of this study

While there has been an increasing number of studies that have used qualitative methods to develop items and levels for questionnaire, study in dental field that has provided sufficient detail to enable an assessment of the rigor of the methods used and patients’ satisfaction replication is rare. The qualitative methods used in this study are comparable to those reported in other qualitative questionnaire studies. The key strength of this study was that it captured a wide range of perspectives from patients in different disciplines. The diversity strengthens the extrapolation of the study’s findings to different specific dental care services. Additionally, sample sizes in qualitative research vary significantly as a result [18]. The sample of 30 was sufficient to develop meaningful items for the satisfaction questionnaire. However, there was a limitation. The study was carried out in a teaching hospital and most of the treatments were completed by the students. The treated patients are for teaching purposes while clinics were non-profit-oriented. Thus, the admission of patient, treatment duration, time needed for each visit and price may vary from the concerns of patients under private settings. These patients may not be a good representation of private dental service and require intensive treatment by specialists.

### Comparison with the DSQ questionnaire

The aspects mentioned during the focus group discussions were compared with sub-themes and items in DSQ. The majority of the items in DSQ were covered by the aspects mentioned in focus group discussions except for five items (see notes of Table 2).

“Attitude” was a main concern when talking about dental satisfaction. Both focus group discussions and DSQ identified the attitude of dentists although the relevant items in DSQ were classified into “Quality”. In the focus group discussion, the participants emphasized more on their dentists’ attention and concern of patients’ suffering, their carefulness in examination, their clarity of explanation about the treatment and following their situation. However, this was not exactly reflected by the wording of the relevant items in DSQ. For example, the wording “thorough” (Table 2, DSQ item 11) may be reminiscent of all the above-mentioned aspects. It is worth considering whether using more specific wordings can enhance the validity of the questionnaire. In addition, the attitude of the dental supporting staff also affects the patient’s satisfaction whereas it is not reflected in the DSQ.

Cost of dental care were both covered in focus groups discussions and DSQ. However, there was one more DSQ item asking whether the dentists tried to reduce unnecessary expense, which was not mentioned in the focus group discussions.

Apart from “Attitude”, “Convenience” as quoted by the participants was another big concern. In DSQ the relevant items were classified into domains of “Access” and “Availability”. However, the difference between “accessibility” and “availability” is subtle in Cantonese and the participants mentioned all relevant aspects when asked about “convenience” (*Is it convenient for you to visit a dentist in PPDH? Why?*). In the DSQ questionnaire, aspects related to accessibility and availability included opening hours, appointment booking, location of hospital, waiting time in the clinic and manpower (whether there is enough dentist or not). In focus group discussion, the participant mentioned more aspects related to convenience such as emergency service, admission of patient, treatment duration, and these aspects were considered because PPDH is a teaching hospital. The inconveniences and long treatment duration of students’ cases could contribute to patient dissatisfaction.

As to pain management, the DSQ items asked more about the patients’ concern towards feeling pain and patients’ avoidance of dental visit due to fear of pain, which were not mentioned in focus groups. However, the participants mentioned more specifically about pain management during treatment and between treatments.

Other than the three aspects that were also covered in DSQ, participants in focus group discussions mentioned more aspects when talking about “Quality”, not only technology and equipment of the hospital, but also infection control of the hospital and perceived skills of the dentists.

Importantly, the patients’ perceived needs for oral disease prevention were expressed by patients when they assessed dental service. The appropriate oral health education provided by dentists, the accessibility of oral health information in the hospital environment and arranged regular follow-up would affect patient satisfaction. It is worth noting that in the focus group discussion, the question “*Dentists should do more to keep you from having problems with your teeth*” was not emerged directly. However, we can regard all the statements of perceived needs as an elaboration of this item and to incorporate it in the future design of DSQ questionnaire would be worthwhile.

### Implication for future dental satisfaction questionnaire development

It would be recommended to improve the content validity by adding these aspects into DSQ, which will result in a new questionnaire. However, patients expressed both negative and positive views about certain satisfaction aspects. The appropriate wording and direction of wording would be an important concern when adding in these new items.

From previous studies, the internal consistency of some domains in DSQ was low. For calculating the satisfaction scores of each domain especially the domain with low Cronbach’s Alpha value may not be as meaningful. The same item may belong to a different domain in original DSQ and focus group study. It is suggested that when using the modified questionnaire, items should be viewed separately first, and then regrouped to find domains with better internal consistency before calculating the scores of each domain.

## Conclusions

The use of semi-structured focus groups helped the derivation of items and themes for the design of a questionnaire by eliciting the views of patients. The new items not covered by DSQ emerged in the focus group, which may suggest areas for further development. As the satisfaction measurement from the qualitative study supported the collection of appropriate and meaningful data, it would assist the dental care system in providing optimal and relevant care to patients.

## Abbreviations

DSQ: Dental satisfaction questionnaire
DSS: Dental satisfaction scale
DVSS: Dental visit satisfaction scale
PPDH: Prince Philip Dental Hospital (Hong Kong)
SERVQUAL: Scale for measuring consumer perception of service quality
